# Limitations of acetaminophen as a reference hepatotoxin for the evaluation of in vitro liver models

**DOI:** 10.1093/toxsci/kfae133

**Published:** 2024-10-14

**Authors:** Lucia A Livoti, Rowena Sison-Young, Dennis Reddyhoff, Ciarán P Fisher, Iain Gardner, Rafael Diaz-Nieto, Christopher E Goldring, Ian M Copple

**Affiliations:** Department of Pharmacology & Therapeutics, Institute of Systems, Molecular & Integrative Biology, University of Liverpool, Liverpool, L69 3GE, United Kingdom; Department of Pharmacology & Therapeutics, Institute of Systems, Molecular & Integrative Biology, University of Liverpool, Liverpool, L69 3GE, United Kingdom; Certara Predictive Technologies, Sheffield, S1 2BJ, United Kingdom; Certara Predictive Technologies, Sheffield, S1 2BJ, United Kingdom; GSK Research and Development Ltd, Stevenage, SG1 2NY, United Kingdom; Certara Predictive Technologies, Sheffield, S1 2BJ, United Kingdom; Liverpool University Hospitals NHS Foundation Trust, Liverpool, L7 8YE, United Kingdom; Department of Pharmacology & Therapeutics, Institute of Systems, Molecular & Integrative Biology, University of Liverpool, Liverpool, L69 3GE, United Kingdom; Department of Pharmacology & Therapeutics, Institute of Systems, Molecular & Integrative Biology, University of Liverpool, Liverpool, L69 3GE, United Kingdom

**Keywords:** acetaminophen, DILI, hepatocytes, bioactivation

## Abstract

Acetaminophen is commonly used as a reference hepatotoxin to demonstrate that in vitro human liver platforms can emulate features of clinical drug-induced liver injury. However, the induction of substantial cell death in these models typically requires acetaminophen concentrations (∼10 mM) far higher than blood concentrations of the drug associated with clinical hepatotoxicity (∼1 mM). Using the cytochrome P450 inhibitor 1-aminobenzotriazole, we show that acetaminophen toxicity in cultured human, mouse, and rat hepatocytes is not dependent on N-acetyl-p-benzoquinonimine formation, unlike the in vivo setting. This finding highlights the limitation of using acetaminophen as a reference hepatotoxin for the evaluation of in vitro liver models. Hence, we make recommendations on the selection of reference hepatotoxins for this purpose.

As the liver is a major target of the toxicity associated with therapeutic drugs and other chemicals (Stravitz and Lee 2019), many in vitro human liver models (e.g., spheroids, organoids, microphysiological systems) have been developed and evaluated with a view to increasing the translational relevance of the nonclinical liver safety assessment process (Weaver et al. 2020). These platforms can incorporate single (e.g., primary hepatocytes) or multiple liver cell types and are often reported to exhibit a more “in vivo-like” phenotype, when compared with traditional in vitro cell lines or hepatocytes cultured in 2D (Fernandez-Checa et al. 2021). We and others have used acetaminophen (APAP), along with other hepatotoxic compounds, to evaluate the performance of selected in vitro human liver models as predictors of drug-induced liver injury (DILI) (Richert et al. 2016; Sison-Young et al. 2017). APAP remains the most common cause of acute liver failure in many countries (Lee 2013) and hence is a seemingly rational reference hepatotoxin. In vivo, APAP hepatotoxicity requires cytochrome P450 (CYP450) mediated bioactivation to the highly reactive N-acetyl-p-benzoquinonimine (NAPQI), which depletes glutathione (GSH) stores, induces oxidative stress, and covalently reacts with hepatocellular proteins leading to necrosis and liver failure (Fig. 1a) (Ramachandran and Jaeschke 2019).

**Fig. 1.**
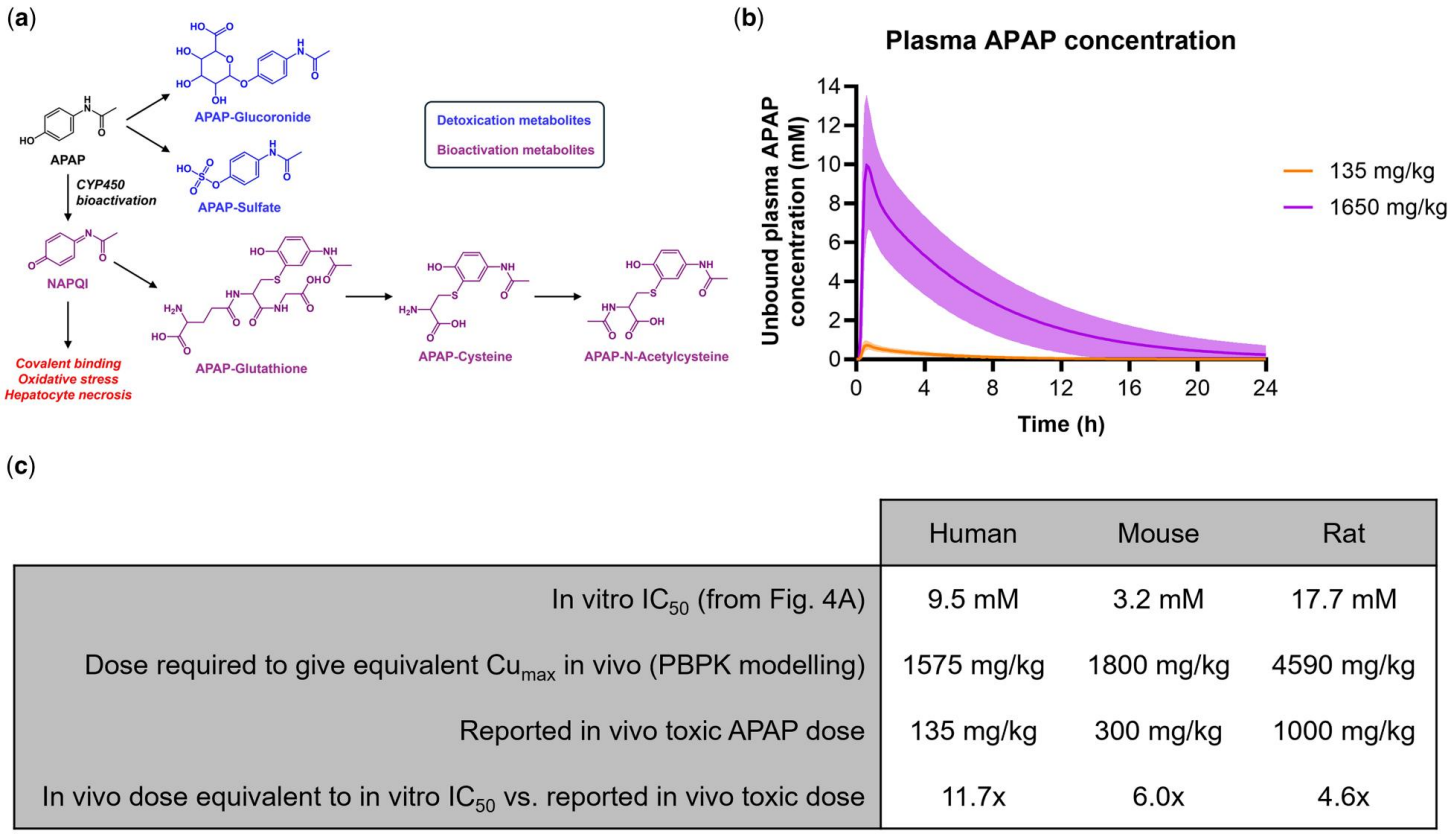
Disconnect in APAP toxicity between in vivo and in vitro contexts. a) Schematic representation of APAP metabolism and hepatotoxicity. b) Simulations of APAP dosing (Simcyp v18 r1; Certara) in humans using a previously published and verified PBPK model (Russomanno et al. 2023). The plot shows the mean simulated unbound plasma concentrations of APAP in fasted healthy volunteers following single oral administration of the indicated doses and population variability (10 trials of 5 males and 5 females aged 20–50 years; shaded area is 95% confidential interval). c) APAP *in vitro* IC_50_ concentrations (from Fig. 4a) and *in vivo* doses required to achieve similar plasma Cu_max_ concentrations, based on PBPK modeling, compared with reported *in vivo* hepatotoxic doses for the 3 species (LiverTox 2012; Russomanno et al. 2023).

An acute hepatotoxic dose of APAP in humans is considered to be >10 g (LiverTox 2012), which equates to >135 mg/kg based on an average body weight of 75 kg. Using physiologically based pharmacokinetic (PBPK) modeling and simulation in a virtual human population, which could reproduce reported clinical plasma concentrations of APAP following a single oral dose (Singla et al. 2012), we have predicted that the maximum unbound plasma concentration (Cu_max_) of APAP expected at a dose of 135 mg/kg is 0.84 ± 0.29 mM (Fig. 1b). This is consistent with the Rumack-Matthew nomogram used in the clinical management of APAP overdose. Indeed, the “200 line” of this nomogram equates to an APAP plasma concentration of 200 µg/ml, or 1.3 mM, at 4-h post-ingestion. This indicates the lowest APAP plasma concentration at which N-acetylcysteine therapy should be administered for early-presenting overdose patients. Therefore, based on this nomogram and our PBPK modeling, liver toxicity is typically observed in the clinic at APAP blood concentrations of ≥1 mM. However, in cultured primary human hepatocytes and many other in vitro human liver models, the concentration of APAP required to induce substantial cell death is typically ≥10 mM (Jemnitz et al. 2008; Xie et al. 2014; Richert et al. 2016; Sison-Young et al. 2017), although it should be noted that these acute studies do not always reflect the clinical time course of APAP hepatotoxicity, which can take several days to manifest (LiverTox 2012). Nevertheless, to achieve a Cu_max_ of 10 mM in humans would require a dose of ∼1,650 mg/kg (i.e., 124 g of APAP for an average body weight of 75 kg), based on PBPK modeling and simulation (Fig. 1b). This indicates that there is a major disconnect between the in vivo and in vitro contexts when modeling APAP toxicity.

The broad-spectrum CYP450 inhibitor 1-aminobenzotriazole (ABT) has been shown to prevent APAP liver injury in mice (Forootan et al. 2017), highlighting the importance of metabolic bioactivation in the mechanism of APAP hepatotoxicity in vivo. In this study, we have used ABT to block conversion of APAP to NAPQI in human, mouse, and rat primary hepatocytes cultured in 2D, and determine the role of metabolic bioactivation in APAP toxicity in vitro. For research involving human hepatocytes, informed consent was obtained from each patient donating tissue as part of planned liver resections. The study was approved by the National Health Service North West-Liverpool Central Research Ethics Committee (11/NW/0327) and was in accordance with both the Declarations of Helsinki and Istanbul. Animal experiments were performed in accordance with a license granted under the UK Animals (Scientific Procedures) Act 1986 and were approved by the University of Liverpool Animal Ethics Committee.

In hepatocytes from all 3 species, ABT completely blocked the formation of APAP bioactivation metabolites (APAP-GSH, APAP-Cys, APAP-NAC), and had little impact on the formation of the glucuronide and sulfate detoxication metabolites (Fig. 2), as expected. ABT afforded some protection against APAP-induced GSH depletion, particularly in mouse and rat hepatocytes at early time points (Fig. 3). However, ABT did not affect the loss of viability of human, mouse, or rat cells following exposure to APAP (Fig. 4). Indeed, in ABT pretreated hepatocytes from all 3 species, there was still a concentration-dependent decrease in cellular ATP content following exposure to APAP for 24 hr (Fig. 4), despite ABT reversing the GSH depletion at earlier time points (Fig. 3), indicative of a bioactivation-independent mechanism of APAP-induced cell death in vitro. The cell viability experiments also confirmed that the in vivo doses predicted to yield Cu_max_ values equivalent to the in vitro IC_50_ concentrations are 5- to 10-fold higher than the reported in vivo toxic doses of APAP (Fig. 1c). Hence, although APAP causes some CYP450-dependent depletion of GSH, the extent of APAP bioactivation is relatively minimal in cultured primary hepatocytes from humans, mice, and rats. This is consistent with previous reports in which APAP-protein adducts were shown to peak at ∼0.15 nmol/mg protein in mouse hepatocytes in vitro (Miyakawa et al. 2015) and at ≥5-fold higher levels following administration of a hepatotoxic dose of APAP in vivo (Nguyen et al. 2021). Therefore, we conclude that APAP toxicity is not dependent on metabolic bioactivation in primary hepatocytes from humans, mice, and rats, in contrast to the in vivo setting.

**Fig. 2.**
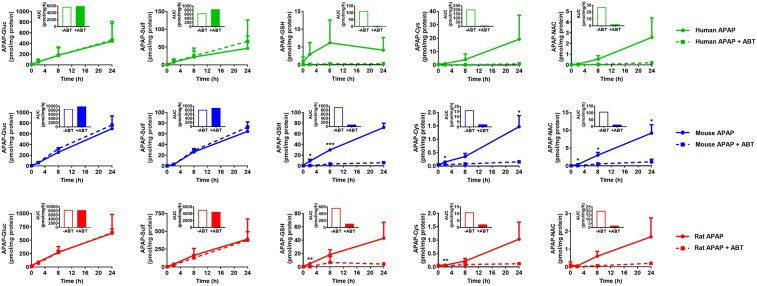
Inhibition of CYP450 activity prevents APAP bioactivation in primary hepatocyte cultures. Fresh primary hepatocyte monocultures were generated from the livers of human donors (Heslop et al. 2017), C57Bl/6J mice (Li et al. 2010), and Sprague Dawley rats (Shen et al. 2012), as previously described. Human cells were isolated 2–3 h following liver resection. Within 1 hr of isolation, cells were seeded onto Collagen-I coated plates and incubated for 3 (mouse and rat hepatocytes) or 16 (human hepatocytes) hr. Unattached cells were then removed, and the remaining cells were pretreated with 1 mM ABT for 1 hr prior to treatment with 10 mM APAP, or media as vehicle control, for the indicated times. Dimethyl sulfoxide, a known CYP450 inhibitor, was not used as a vehicle in these experiments. APAP metabolites were quantified using LC-MS/MS and deuterated standards. Insets show the area under the curve (AUC). Mean+SD. (*n* = 3). Significance determined by unpaired *t*-test, *P ≤ 0.05, **P ≤ 0.01, ***P ≤ 0.001.

**Fig. 3.**
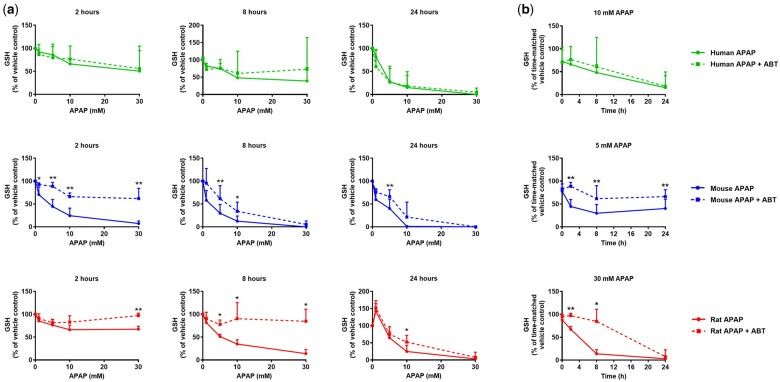
Inhibition of CYP450 activity prevents GSH depletion by APAP in rodent primary hepatocyte cultures. As described in Fig. 2, primary hepatocyte monocultures were pretreated with 1 mM ABT for 1 hr prior to treatment with APAP for up to 24 hr. Cellular GSH content was determined, as previously described (Vandeputte et al. 1994), in cells exposed to (a) the indicated concentrations of APAP for 2, 8, or 24 hr, or (b) approximate IC_50_ concentrations (human 10 mM, mouse 5 mM, rat 30 mM) of APAP for the indicated times. Mean+SD (human *n* = 3, mouse and rat *n* = 5). Significance determined by unpaired *t*-test, *P ≤ 0.05, **P ≤ 0.01.

**Fig. 4.**
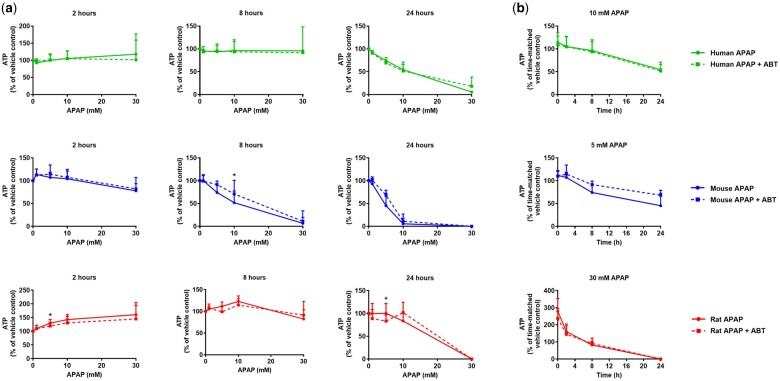
Inhibition of CYP450 activity does not prevent APAP toxicity in primary hepatocyte cultures. As described in Fig. 2, primary hepatocyte monocultures were pretreated with 1 mM ABT for 1 hr prior to treatment with APAP for up to 24 hr. Cellular ATP content was determined, as previously described (Sison-Young et al. 2017), in cells exposed to (a) the indicated concentrations of APAP for 2, 8, or 24 hr, or (b) approximate IC_50_ concentrations (human 10 mM, mouse 5 mM, rat 30 mM) of APAP for the indicated times. Mean+SD (human *n* = 3, mouse and rat *n* = 5). Significance determined by unpaired *t*-test, *P ≤ 0.05.

Our finding that ABT is unable to protect against the loss of cell viability associated with a range of APAP concentrations in mouse, rat, and human hepatocytes is consistent with a previous report using primary mouse cells (Miyakawa et al. 2015). In this study, APAP-protein adducts (a surrogate marker of APAP bioactivation) increased in a concentration-dependent manner up to 5 mM APAP and then plateaued, despite higher APAP concentrations causing more substantial cell death (Miyakawa et al. 2015). The authors of this study reported that ABT exhibited a completely protective effect only at APAP concentrations of ≤5 mM, with only partial protection observed at higher APAP concentrations. Taken together with our findings across 3 species, this strengthens the evidence that a bioactivation-independent mechanism is a key driver of APAP toxicity in vitro, in contrast to the in vivo setting.

Although the clinical antidote for APAP overdose, N-acetylcysteine, has been shown to protect against APAP toxicity in primary hepatocyte cultures and other in vitro liver models (Xie et al. 2014), this effect has not been proven to involve the suppression of reactive metabolite-mediated insult. Other studies have demonstrated that compounds known to nonspecifically inhibit CYP450 enzymes can protect against APAP toxicity in primary hepatocytes, albeit typically without accompanying evidence for the suppression of APAP bioactivation under the reported experimental conditions (Anundi et al. 1993; Xie et al. 2013). The ability of CYP450 inhibitors to protect against APAP toxicity in primary hepatocyte cultures may be influenced by species differences in the degree of APAP bioactivation. For example, compounds known to inhibit CYP450 enzymes have been shown to protect against APAP toxicity in primary hepatocytes isolated from hamsters (Harman and Fischer 1983), which are very sensitive to APAP liver injury in vivo due to the relatively high extent of reactive metabolite generation (Davis et al. 1974). Yet, in primary hepatocytes isolated from less sensitive and more commonly used species such as humans, mice, and rats, along with other in vitro liver models, it is likely that the loss of viability observed following APAP exposure is at least partly a nonspecific effect related to the high concentrations of the drug required to induce overt cell death. It has been reported that APAP is able to inhibit mitochondrial function in HepG2 cells and platelets at the mM concentrations required to kill primary hepatocytes in vitro (Kamalian et al. 2015; Beţiu et al. 2024), despite the former cell types exhibiting substantially less CYP450 bioactivation capacity than hepatocytes in vivo. Further work is required to elucidate the exact mechanism of APAP toxicity at the relatively high concentrations required to kill primary hepatocytes in vitro.

In light of the above findings, we caution against using APAP as a single agent for assessing the toxicological relevance of an in vitro liver model, particularly when concentrations >1 mM are required to induce overt cell death. To fully demonstrate that an in vitro liver model replicates the in vivo mechanism of APAP toxicity, it is important to show that ABT (or an alternative, specific inhibitor of the relevant CYP450 enzymes) blocks both the formation of bioactivation metabolites and cell death following exposure to APAP. Given the relative uniqueness of APAP liver injury (i.e., requirement for a large dose and the formation of substantial quantity of reactive metabolite, alongside the extensive depletion of GSH stores, in order to induce hepatocellular injury) it is prudent to consider other intrinsic hepatotoxins as alternative reference compounds when evaluating in vitro liver models. The ProEuroDILI consortium recently provided an evidence-based, consensus-driven list of reference drugs for validation of in vitro models of DILI, which notably excluded APAP (Segovia-Zafra et al. 2024). Other consortia have recommended compounds that cause hepatocellular injury via a range of mechanisms and can be assessed alongside less toxic comparators to determine the specificity of the test system (Dragovic et al. 2016; Baudy et al. 2020). Example drug pairs include clozapine/olanzapine, tolcapone/entacapone, and troglitazone/pioglitazone. As an absolute minimum, it should be demonstrated that a DILI compound causes loss of viability of the cellular components of an in vitro liver platform at concentrations that are relevant in the context of clinical pharmacokinetic data, and that the comparator compound is substantially less toxic. Ideally, multiple DILI compounds should be evaluated, and, where feasible, the recapitulation of key in vivo mechanisms should be confirmed in the in vitro setting.

These simple recommendations complement existing broader guidelines on the development of in vitro liver platforms in the context of drug safety assessment (Baudy et al. 2020). Taken together, we believe that these approaches can improve the robustness of benchmarking for in vitro liver models, and ultimately support the development of translationally relevant and practical tools to underpin human liver research and facilitate the nonclinical evaluation of new medicines. Furthermore, as progress is made on the incorporation of relevant immune system components into in vitro liver platforms, the ability to identify drug candidates with a risk of idiosyncratic, immune-mediated DILI will also improve. In addition to the intrinsic hepatotoxins noted above, reference compounds encompassing immune mechanisms will become more relevant in this context. In the characterization of novel, and increasingly complex, in vitro platforms, mathematical modeling and simulation have an important role to play. The use of such an approach to understand in vitro biokinetics (Fisher et al. 2019), aid in vitro to in vivo extrapolation (e.g., PBPK modeling), and investigate cross-species translation (Russomanno et al. 2023) should be considered alongside the recommendations outlined above. We look forward to further advances in this exciting area of research over the coming years.
